# Corticosterone after early adolescent stress prevents social avoidance, aversive behavior, and morphine-conditioned place preference in adulthood

**DOI:** 10.1007/s00213-024-06616-7

**Published:** 2024-05-28

**Authors:** Samantha O. Vanderhoof, Carly J. Vincent, Jasmin N. Beaver, Maeson S. Latsko, Ricardo Aguilar-Alvarez, Aaron M. Jasnow

**Affiliations:** 1 Department of Psychological Sciences, Brain Health Research Institute, Kent State University, Kent, USA; 2 Department of Pharmacology, Physiology, and Neuroscience, University of South Carolina School of Medicine, Columbia, USA

**Keywords:** Fear conditioning, Foot-shock stress, Social defeat, Drug-seeking

## Abstract

**Rationale:**

Stress during childhood or adolescence increases vulnerability to psychiatric disorders in adults. In adult rodents, the delayed effects of stress can increase anxiety-like behavior. These effects, however, can be prevented with post-stress administration of corticosterone (CORT). The effectiveness of CORT in preventing adolescent stress-induced emotional behavior alterations in adulthood has yet to be investigated.

**Objectives:**

Here, we investigated the interactions between early adolescent stress and exogenous corticosterone on adult social, aversive, and drug-seeking behavior in mice, which are translationally related to symptoms associated with psychiatric and substance abuse disorders.

**Methods and results:**

A single administration of CORT in drinking water (400ug/mL) for 24 h after social defeat or context fear conditioning prevents defeat-induced social avoidance, alters fear processing, prevents adolescent stress-induced anhedonia, and prevents stress-potentiated morphine place preference in adulthood. Exogenous CORT did not immediately prevent stress-induced potentiation of morphine conditioned-place preference in adolescents but did so in adult mice. However, when administered to adolescent mice, CORT also prevented the incubation of morphine-conditioned place preference into adulthood. Lastly, exogenous CORT administration blunted endogenous corticosterone but was unrelated to freezing behavior during a fear test.

**Conclusions:**

This is the first demonstration of adolescent post-stress CORT promoting socio-emotional resilience and preventing drug-seeking behavior. Our data suggest elevated corticosterone after a stress experience promotes resilience for at least 40 days across the developmental transition from adolescence to adulthood and is effective for socio-emotional and drug-seeking behavior. These results are critical for understanding how adolescent stress impacts emotional and drug-seeking behavior into adulthood.

## Introduction

Traumatic experiences during childhood or adolescence are highly predictive of psychiatric disorder diagnoses such as anxiety, depression, and substance use disorder (Kessler et al. 2010). In rodents, stress during or around adolescence uniquely affects hypothalamic-pituitary-adrenal axis function, behavior, and gene expression (Lui et al. 2012; Romeo et al. 2007, 2008, 2014, 2016). We previously found that adult mice exposed to social defeat during early adolescence (P31-P33) displayed two social phenotypes; one phenotype avoided social interaction with novel mice while the other exhibited typical social-approach behavior (Latsko et al. 2016). Interestingly, the social effects of defeat were not observed in early adolescence – the mice showed typical social behavior – but a subset of mice had increased circulating corticosterone. This increased corticosterone was positively correlated with a resilient social phenotype in adulthood: high corticosterone-secreting adolescent mice continued to approach novel mice similar to non-defeated controls.

The above finding suggests that elevated endogenous corticosterone after stress may confer some resilience to adolescent stress. This agrees with clinical reports suggesting a link between trauma during childhood and adolescence and low cortisol reactivity in adulthood (Heim et al. 2001; Hinkelmann et al. 2013; Yehuda et al. 2001). Moreover, our results agree with other reports of exogenous corticosterone protecting against the delayed effects of stress (Chakraborty et al. 2020; Magarinos et al. 1998; Rao et al. 2012). Exogenous corticosterone in adult rodents prevents the anxiety-inducing effects of acute immobilization stress. It is thought that corticosterone prevents anxiety-like behavior by preventing increased spine numbers on basolateral amygdala principal neurons (Chakraborty et al. 2020). However, several important but unanswered questions are raised by these previous studies: (1) how long do the protective effects of corticosterone last; (2) do the effects apply to different developmental periods; and (3) do they extend beyond anxiety-related behavior? The studies described here investigate interactions between early adolescent stress and exogenous corticosterone on adult social, aversive, and drug-seeking behavior in mice, which are translationally related to symptoms associated with psychiatric and substance abuse disorders.

## Materials and methods

### Animals

All experiments used C57BL/6J male mice, postnatal day 29 (P29) or older, generated from a breeding colony in the Department of Psychological Sciences at Kent State University or a breeding colony at the University of South Carolina School of Medicine. All mice were filial generation 5 or less. Mice were group-housed until social defeat or fear conditioning, then single-housed thereafter with free access to food and water in a room maintained on a 12:12 light/dark cycle. This was done to match our previous study (Latsko et al. 2016), and to be able to monitor fluid intake. All procedures were conducted in a facility accredited by the AALAC, per the NIH guidelines, and with approval by the Kent State University and University of South Carolina IACUC.

### Behavioral procedures

#### Repeated social defeat

Social defeat involved placing experimental mice (P30-31) into the home cage of an aggressive, territorial CD-1 mouse for five minutes or until three attacks were observed– whichever came first, as described previously (Latsko et al. 2016). Following exposure to attacks, mice were separated across a Plexiglas divider for the rest of the hour in the CD-1 mouse cage. After the 55-minute separation, mice were transferred into the cage of a novel CD-1 mouse. The defeat was repeated until each mouse encountered four different aggressors per day for two consecutive days, totaling eight defeats by eight separate aggressors. Control mice were placed in home cages of eight different CD-1 mice – one hour per cage, four cages per day, over two consecutive days – without the aggressor present. Social defeat and control procedures occurred between zeitgeber (ZT) 4 and ZT8 – with ZT0 indicating lights on and ZT12 indicating lights off.

#### Social interaction

Experimental mice were tested in social interaction 24 hours (ZT8) after the last social defeat and within four hours of the onset of the dark cycle (P32). These experimental mice were tested again 30 days (delayed) after the initial interaction test (P62). During each social interaction test, mice were placed in an open field arena (Coulbourn Instruments) where social behavior was measured under dim lighting. A polyvinyl chloride (PVC) pipe (10” H x 3.5” D) was placed in the corner of the open field arena with an opening covered by wire mesh (4.5” W x 2” H) for placement of a novel, age-matched, nonaggressive CD-1 mouse. During the first trial, experimental mice were placed in the center of the arena and allowed to explore for 300 s without the presence of the CD-1 mouse. Immediately following the first trial, social targets were placed in the PVC pipe, and experimental mice were again allowed to explore for 300 s. Time spent interacting with social targets was measured via ANY-maze 4.99 software (Stoelting, Wood Dale, IL).

#### Conditioned place preference

Conditioned place preference (CPP) was performed in four identical conditioning chambers. Each CPP apparatus consisted of a polycarbonate enclosure that contained three compartments. Two compartments are of identical dimensions (17 × 12.5 × 19 cm H), while the third compartment (8 × 13 × 19 cm H) is a neutral chamber. All three compartments have distinct contextual cues, including varied wall patterns and floor textures, and can be individually closed off using removable sliding doors. Enclosures were cleaned with a 70% ethanol solution before use each day and between conditioning andtesting rounds. The entire place preference paradigm lasted ten days. Animals started conditioning 24-hours following the final social interaction test or fear testing. On the first day (habituation), mice were placed into the neutral chamber of the apparatus with free access to all chambers for 15 min. Initial preference was calculated, and mice were counter-conditioned against the initial preference. On subsequent training days, mice were given subcutaneous injections of morphine or saline and placed into the neutral chamber, they crossed over to the respective assigned test chamber paired with that treatment. If mice did not cross readily, the experimenter guided the mice into the required chamber. Mice received a total of 4 saline pairings and 4 morphine parings. During these conditioning sessions, mice were confined to the respective chambers to form an association between the drug-induced state and contextual cues of the apparatus for 30 min. The chamber placement alternated with the substance being injected. Twenty-four hours after the last training session, mice were tested for acquisition of conditioned place preference in the absence of drug. The final preference for one chamber was calculated in seconds (test time morphine–test time saline)–(habituation time morphine–habituation time saline). Mice were excluded if initial preference exceeded 75%. Conditioned place preference occurred between ZT3 and ZT5.

#### Context fear conditioning (foot-shock stress) and testing

Fear conditioning served as foot-shock stress and allowed us to assess subsequent freezing responses and was performed in four identical conditioning chambers (7”W x 7”D x 12”H) containing two Plexiglas walls, two aluminum sidewalls, and a stainless steel grid-shock floor (Coulbourn Instruments, Allentown, PA). The training context consisted of the conditioning chamber with a polka-dot insert attached to the rear Plexiglas wall, white noise (70db), dim illumination, and the stainless-steel grid floors were cleaned with 70% ethanol. Mice were pre-exposed twice to the context for five minutes beginning two days before fear conditioning. Fear conditioning (referred to as Shock in the figures) occurred in the training context with five footshocks (1s, 1.0 mA), separated by 90s inter-shock intervals (ISI). Mice were removed from the apparatus 30 s after the last shock and returned to their home cage. Control mice (referred to as No shock) underwent identical procedures except for the shock administration. Percent freezing was calculated, and mice were sorted using match-random sampling based on the final 30 s of freezing on the training day. Twenty-four hours (immediate) and 30 days (delayed) after training, we conducted a 3-minute test in the training context. Percent time freezing was calculated by Freezeframe Software (Actimetrcis, Wilmette, IL). All fear conditioning procedures occurred between ZT8 and ZT9.

#### Sucrose preference

The sucrose consumption tests were performed using a two-choice test. Mice had free access to water and a sucrose solution using standard water bottles and sipper tubes for 24 h, within the animal’s home cage. They were first trained to consume water in the two bottles. The next day, sucrose consumption tests began: a bottle filled with a 2% sucrose solution replaced a bottle of water for 24 h. After 12 h, the water bottles were switched to account for a side preference. Consumption was measured at both 12 h and 24 h. Sucrose preference was calculated as follows ($\frac{Sucrose \,Solution\, Intake \left(mL\right)}{Total \,Fluid \,Intake \left(mL\right)}$).

### Drug preparation and administration

Corticosterone (CORT) or vehicle was administered immediately after the second day of social defeat or after context fear conditioning via drinking water in standard water bottles (400 ug/mL in 2.4% ethanol, (Chakraborty et al. 2020; Magarinos et al. 1998; Rao et al. 2012) for 24 h. CORT water bottles were replaced with standard drinking water after 24 h. Morphine sulfate salt pentahydrate (10 mg/kg) (Dong et al. 2006; Ribeiro Do Couto et al. 2006) or saline was administered via subcutaneous injection immediately before mice were placed in the CPP apparatus.

### Corticosterone measurement

All trunk blood samples were collected 30 min after context fear testing, within a 30-minute time window, and within four hours of the onset of the dark cycle to control for circadian variation in corticosterone. CORT treatment was removed from the cages and replaced with drinking water 30 min before context testing to reduce assessing exogenous corticosterone in the bloodstream. Blood samples were allowed to clot for one hour at room temperature, then centrifuged at 2,000 g for one hour at 4 °C. Blood serum was stored at -80 °C until analysis. Serum corticosterone was measured in duplicate using Enzo Life Sciences corticosterone enzyme-linked immunosorbent assay (ELISA) kits (Farmingdale, NY) according to the manufacturer’s instructions. The plate was read at 405 nm with correction at 595 nm. The cross-reactivity for the corticosterone assay was 28.6% for deoxycorticosterone, 1.7% for progesterone, and < 0.3% for all other hormones. The inter-assay variability was < 14%. The sensitivity of the assay was 27pg/mL.

### Statistical analysis

Statistical tests were two-tailed, and significance was set at *P* < 0.05. Data were analyzed using unpaired t-tests and two-way ANOVAs using Tukey or Šidák’s multiple comparison tests across vehicle and CORT-treated groups or between specific groups. All statistical analyses were performed using GraphPad Prism version 10.

### Experiment 1

In Experiment 1, adolescent mice were subjected to social defeat or a control condition on P30 and P31 (ZT4-8) and administered CORT in their drinking water, beginning at ZT8 until social interaction testing at ZT8 the next day (P32). Mice were given normal drinking water for the remainder of the experiment. Mice were left undisturbed except for daily cage checks and weekly cage changes until P62, when they underwent a second social interaction test in adulthood. The next day (P63), mice began morphine-conditioned place preference training and then underwent a place preference test in the absence of drug on P73.

### Experiment 2

In Experiment 2a, we used context fear conditioning during adolescence as an alternative stress procedure (foot shock stress). Adolescent mice were subjected to context fear conditioning or context exposure in the absence of shock on P31 (ZT4-8). Mice were administered CORT in their drinking water from ZT8 on P31 to ZT8 on P32 when they underwent a context fear test in the training context. Mice were given normal drinking water for the remainder of the experiment and were left undisturbed except for daily cage checks and weekly cage changes until another context fear test when they were adults at P62 (ZT8). On P63, mice began morphine-conditioned place preference training and then underwent a place preference test in the absence of drug on P73. Mice then underwent a sucrose preference test.

In Experiment 2b, we utilized a procedure in which mice were tested for fear expression in the original training context or a novel distinct context. Adolescent mice were subjected to context fear conditioning or context exposure in the absence of shock on P31 (ZT4-8). Mice were administered CORT in their drinking water from ZT8 on P31 to ZT8 on P32 when they underwent a context fear test in the training context (Context A) or a novel context (Context B). Mice were given normal drinking water for the remainder of the experiment and were left undisturbed except for daily cage checks and weekly cage changes until another context fear test when they were adults at P62 (ZT8) in the same context they were tested in as adolescents.

### Experiment 3

In Experiment 3a, we tested if CORT administration blocks stress-induced effects in adult mice. Adult mice were subjected to context fear conditioning or context exposure in the absence of shock on P61 (ZT4-8). Mice were administered CORT in their drinking water from ZT8 on P61 to ZT8 on P62 when they underwent a context fear test in the training context. Mice were given normal drinking water for the remainder of the experiment. On P63, mice began morphine-conditioned place preference training and then underwent a place preference test in the absence of drug on P73. Mice then underwent a sucrose preference test.

In Experiment 3b, we aimed to identify if CORT administration reduced stress-potentiated morphine place preference rapidly in adolescence. Adolescent mice were subjected to context fear conditioning or context exposure in the absence of shock on P31 (ZT4-8). Mice were administered CORT in their drinking water from ZT8 on P31 to ZT8 on P32 when they underwent a context fear test in the training context. Mice were given normal drinking water for the remainder of the experiment. On P33 mice began morphine-conditioned place preference training and then underwent a place preference test in the absence of drug on P43. Mice were left undisturbed except for daily cage checks and weekly cage changes until P73, when mice underwent a second conditioned place preference test in the absence of drug.

### Experiment 4

In Experiment 4, we wanted to identify processes mediating the interaction between stress exposure and corticosterone on fear expression. Adolescent mice were subjected to context fear conditioning or context exposure in the absence of shock on P31 (ZT4-8). Mice were administered CORT in their drinking water from ZT8 on P31 to ZT8 on P32. Half of the mice were tested for context fear and then 30 min later were euthanized for blood collection. The other half of the mice were given normal drinking water and were left undisturbed except for daily cage checks and weekly cage changes until they were tested for context fear in the training context on P62. Thirty minutes after the context test, the mice were euthanized, and blood was collected. Serum was isolated and analyzed for corticosterone.

## Results

### Experiment 1: post-stress CORT in adolescence prevents defeat-induced social avoidance and stress-potentiated morphine place preference in adulthood

**Fig. 1:**
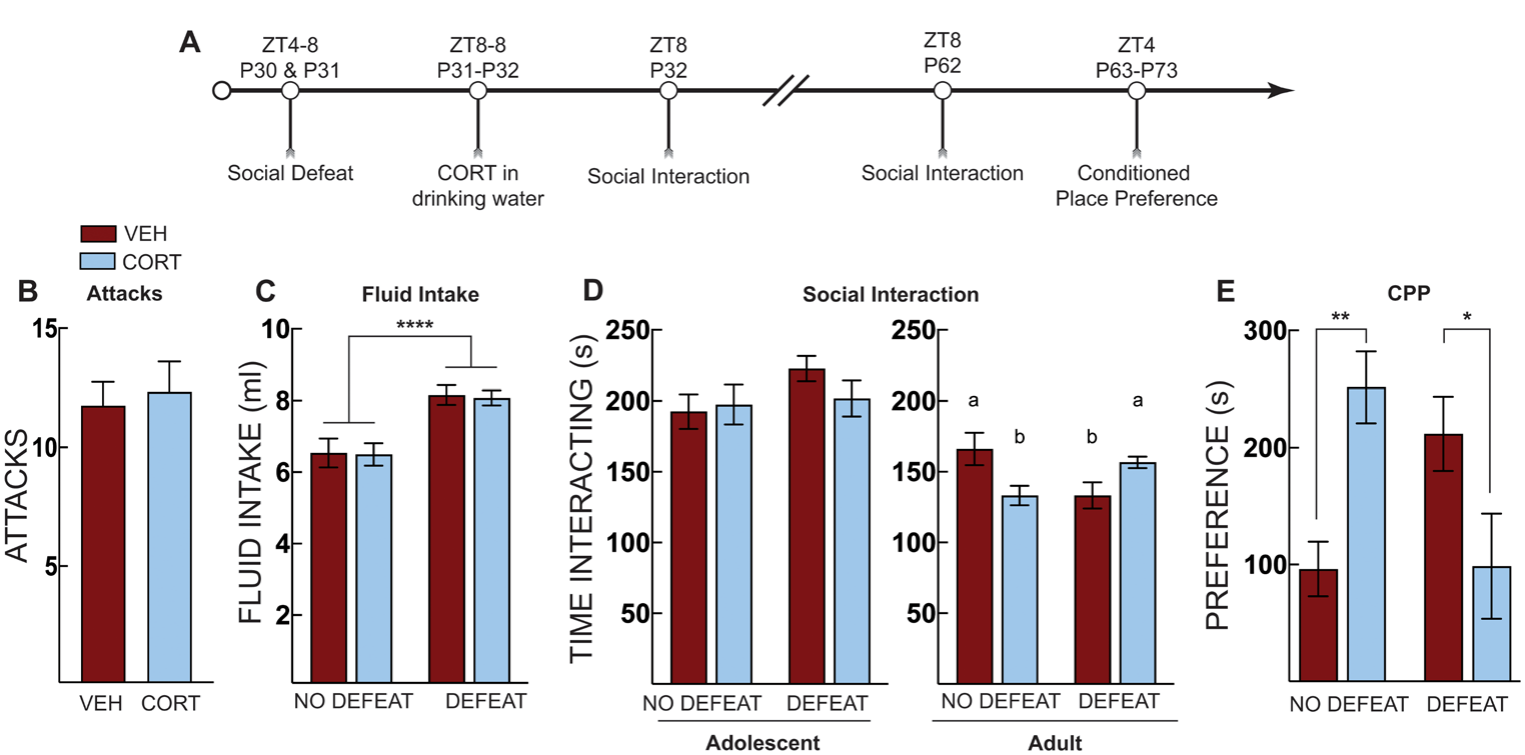
Post-stress CORT prevents defeat-induced social avoidance and stress-potentiated morphine place preference. (**A**) Timeline for adolescent social defeat and corticosterone treatment on social interaction and morphine conditioned place preference. (**B**) Total number of attacks for mice that underwent social defeat stress, there was no difference in total number of attacks between vehicle (VEH, Red Bars) and corticosterone (CORT, Blue Bars)-treated mice. (**C**) Total fluid intake over the 24 h treatment period; defeated mice consumed more water than non-defeated controls. (**D**) Social interaction test in adolescence (P32) and adulthood (P62). There were no immediate effects of social defeat or CORT administration during the P32 test. When mice were tested as adults (P62) CORT or social defeat alone decreased social interaction compared to non-defeated vehicle treated controls. In defeated mice, CORT treatment normalized adult social interaction comparable to non-defeated vehicle controls. (**E**) Morphine conditioned place preference in adulthood; both corticosterone and social defeat alone increased preference for the morphine paired chamber but corticosterone treatment after social defeat prevented this potentiated preference, producing preference scores comparable to non-defeated vehicle treated controls. No defeat VEH (*n* = 13), No defeat CORT (*n* = 10), Defeat VEH (*n* = 13), Defeat CORT (*n* = 13). Bars with different letters indicate a significant difference between treatment groups, *p* < 0.05. Two-way ANOVA with Tukey’s or Sidaks multiple comparison tests. * = *p* < 0.05; ** = *p* < 0.01; **** = *p* < 0.0001

In Experiment 1, adolescent mice were subjected to social defeat and then given CORT until social interaction testing (Fig. 1A). Vehicle (*n* = 12) and CORT-treated (*n* = 12) mice that underwent defeat experienced an equal number of attacks [t (22) = 0.362, *p* = 0.721] (Fig. 1B). We measured CORT and vehicle fluid intake as an indirect measure of total CORT intake. Defeated mice consumed modestly more corticosterone and vehicle fluid than non-defeated mice (Fig. 1C) (Two-way ANOVA, main effect of defeat: F [1, 47] = 26.8, *p* < 0.001), likely due to the increased activity during the defeats. There was no difference in social interaction regardless of treatment or defeat when mice were tested at P32 (Two-way ANOVA, no main effects or interactions) (Fig. 1D). However, when mice were tested at P62, there was a significant defeat X treatment interaction (F [1, 45] = 10.77, *p* = 0.002) (no significant main effects). Defeat reduced social interaction in vehicle-treated mice (*p* = 0.04), as did CORT treatment alone (*p* = 0.04) (Fig. 1D). CORT given to defeated mice, on the other hand, increased social interaction comparable to non-defeated vehicle-treated mice in adulthood (Fig. 1D). These data suggest that corticosterone in early adolescence alters social-approach behavior and promotes resilience to the effects of stress. Thus, CORT administration prevented the defeat-induced social avoidance normally observed in adulthood, as we reported previously (Latsko et al. 2016).

After adult social interaction testing on P62, mice underwent morphine place preference conditioning and were tested for place preference on P72 (Fig. 1A). Early adolescent social defeat potentiated morphine place preference in adulthood; however, CORT reduced this preference for defeated mice only (defeat x treatment interaction: F [1, 45] = 15.26, *p* < 0.001) (Fig. 1E). We compared treatment in the defeated and non-defeated groups using Šidáks multiple comparison test. In non-defeated mice, CORT administration potentiated morphine-place preference (*p* = 0.007). However, in defeated mice, CORT administration significantly reduced morphine place preference (*p* = 0.039). These results agree with previous reports demonstrating that CORT administration alone increased anxiety-like behavior and decreased social behavior (Chakraborty et al. 2020). Further, they suggest that acute social stress or a single overnight administration of CORT during early adolescence has long-term effects on adult appetitive behavior. When CORT is paired with stress, however, it prevents stress-induced potentiation of morphine place preference.

### Experiment 2: post-stress CORT in adolescence prevents generalized fear and stress-potentiated morphine place preference in adulthood

**Fig. 2:**
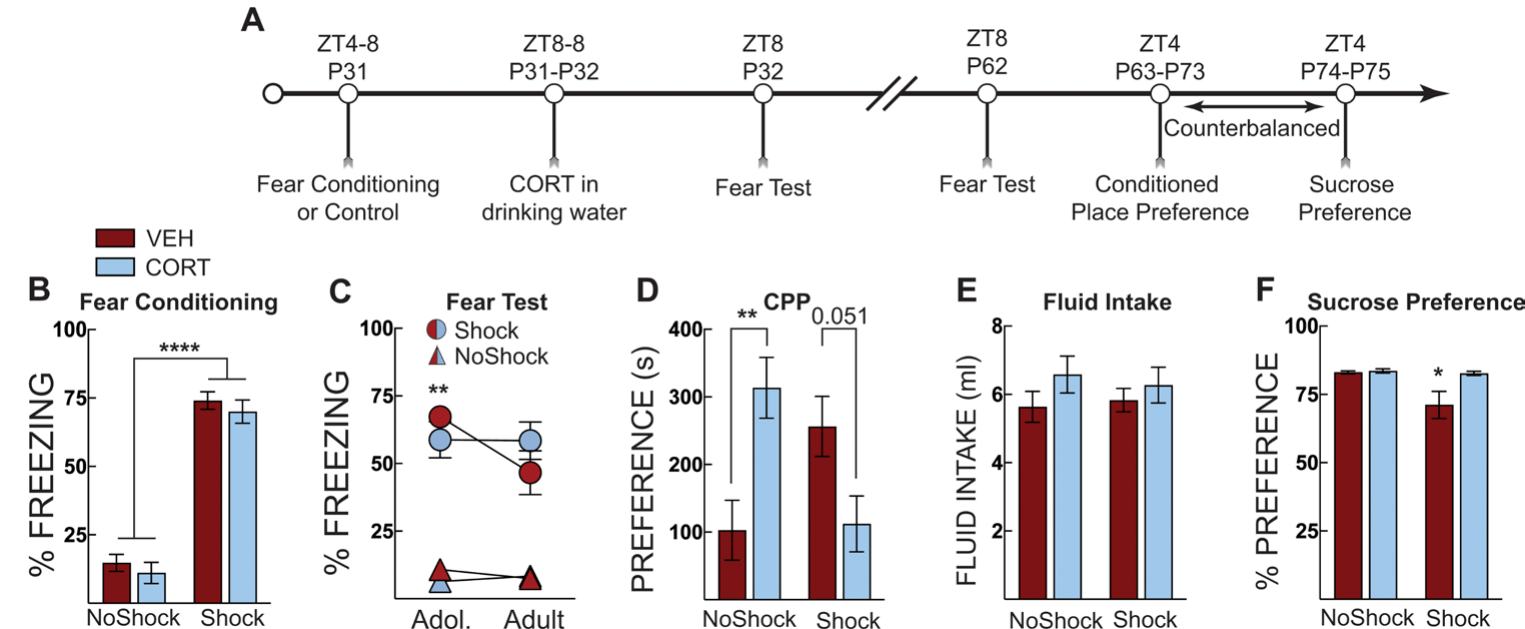
Post-stress CORT maintains context memory specificity and prevents stress-potentiated morphine place preference. (**A**) Timeline for adolescent fear conditioning and CORT treatment on fear expression, morphine place preference and sucrose preference. (**B**) Freezing during context fear conditioning. There was no difference in fear acquisition (average of post-shock freezing across the 5 shocks) between vehicle-treated (VEH, Red Bars) and corticosterone (CORT, Blue Bars)-treated groups. Shocked mice displayed increased freezing compared to no shock control mice. (**C**) During a fear test in adolescence (Adol. P32) only mice exposed to fear conditioning (Circles) had high levels of freezing whereas no shock control groups (Triangles) did not exhibit freezing. VEH-treated mice had a significant reduction in freezing from the adolescent (P32) to adult (P62) test, but when mice were given CORT, this effect was eliminated. No other comparisons were significantly different. (**D**) Morphine conditioned place preference testing identified that CORT alone, and shock alone potentiated morphine place preference compared to no shock control mice administered VEH (though the increase in the VEH-treated group was no statistically significant). Corticosterone prevented the stress-potentiated morphine place preference; preference was no different than no shock VEH-treated controls. (**E**) Total fluid intake over the 24 h period, there was no effect of shock or treatment on fluid consumption. (**F**) Sucrose preference testing identified that shock exposure (in the absence of CORT) produced lower levels of sucrose preference compared to no shock controls and no shock CORT-treated mice. CORT prevented the reduced sucrose preference produced by adolescent stress. No shock VEH (*n* = 11), No shock CORT ( *n* = 12), Shock VEH (*n* = 11), Shock CORT (*n* = 11). Three-way ANOVA with Sidak’s post hoc tests across treatment groups and age (C) and two-way ANOVA with Sidak’s MC post hoc test in (D), * = *p* < 0.05; ** = *p* < 0.01; **** = *P* < 0.0001

In Experiment 2a, forty-six adolescent mice underwent context fear conditioning as a foot shock stress procedure and then received vehicle or CORT in the drinking water until fear testing the next day (Fig. 2A). One mouse was removed due to very high baseline freezing before shock. During fear conditioning, only shocked mice displayed elevated freezing with no differences between treatment groups in their respective training conditions (Two-way ANOVA; main effect of shock: F [1, 41] = 258.6, *p* < 0.0001) (Fig. 2B).

During fear testing (Fig. 2C), we found that only shocked mice had high levels of freezing to the context (Three-way ANOVA; main effect of shock: F [1,40] = 203.9, *p* < 0.0001, and shock x age x treatment interaction: F [1, 40] = 4.167, *p* = 0.048). As fear-conditioned mice aged, there was a significant reduction in fear in the vehicle-treated mice but not in CORT-treated mice (age x treatment interaction: F [1, 40] = 9.04, *p* = 0.0046) (Fig. 2C). This suggests that developmental-dependent reductions in fear memory can be blocked by adolescent CORT treatment. No other comparisons were significantly different.

Following the adult context fear test, mice underwent morphine place preference conditioning. Five mice were removed from the analysis because they had an initial preference greater than 75%. One mouse escaped from the chamber and was removed from the study, leaving 40 mice in the analysis. We found that CORT had different effects on each conditioning group (treatment x shock group interaction: F [1, 36] = 16.37, *p* < 0.001) (Fig. 2D). There were no main effects of shock or treatment alone. We compared treatment in the fear-conditioned (shocked and control (no shock) groups using Šidáks multiple comparison test. In control mice, CORT administration potentiated morphine-place preference (*p* = 0.003). However, in fear-conditioned mice, CORT administration reduced morphine place preference but this was not statistically significant (*p* = 0.051). CORT in the absence of stress did not affect freezing, suggesting adolescent corticosterone has a strong influence on adult reward-related behavior. Importantly, these results replicate the effect of social defeat using a different stress procedure. We attribute the lack of significant post hoc comparisons to high variability in morphine-conditioned place preference despite large differences among the groups. We observed no effects of fear conditioning or treatment on CORT or vehicle fluid intake (Fig. 2E).

Counterbalanced with morphine place preference, we also measured sucrose preference (Fig. 2F). We found fear conditioning (shock) reduced sucrose preference, but this was normalized by CORT treatment (main effect of shock: F [1, 40] = 6.23, *p* = 0.017, main effect of treatment: F [1, 40] = 5.49, *p* = 0.024, shock x treatment interaction: F [1,40] = 4.63, *p* = 0.038). This interaction suggests that stress during early adolescence reduces sucrose preference in adulthood, an indication of anhedonia, which exogenous CORT can alleviate. This finding also provides evidence that the reduced morphine place preference in CORT-treated and shocked mice was not due to a general reduction in reward seeking.

**Fig. 3:**
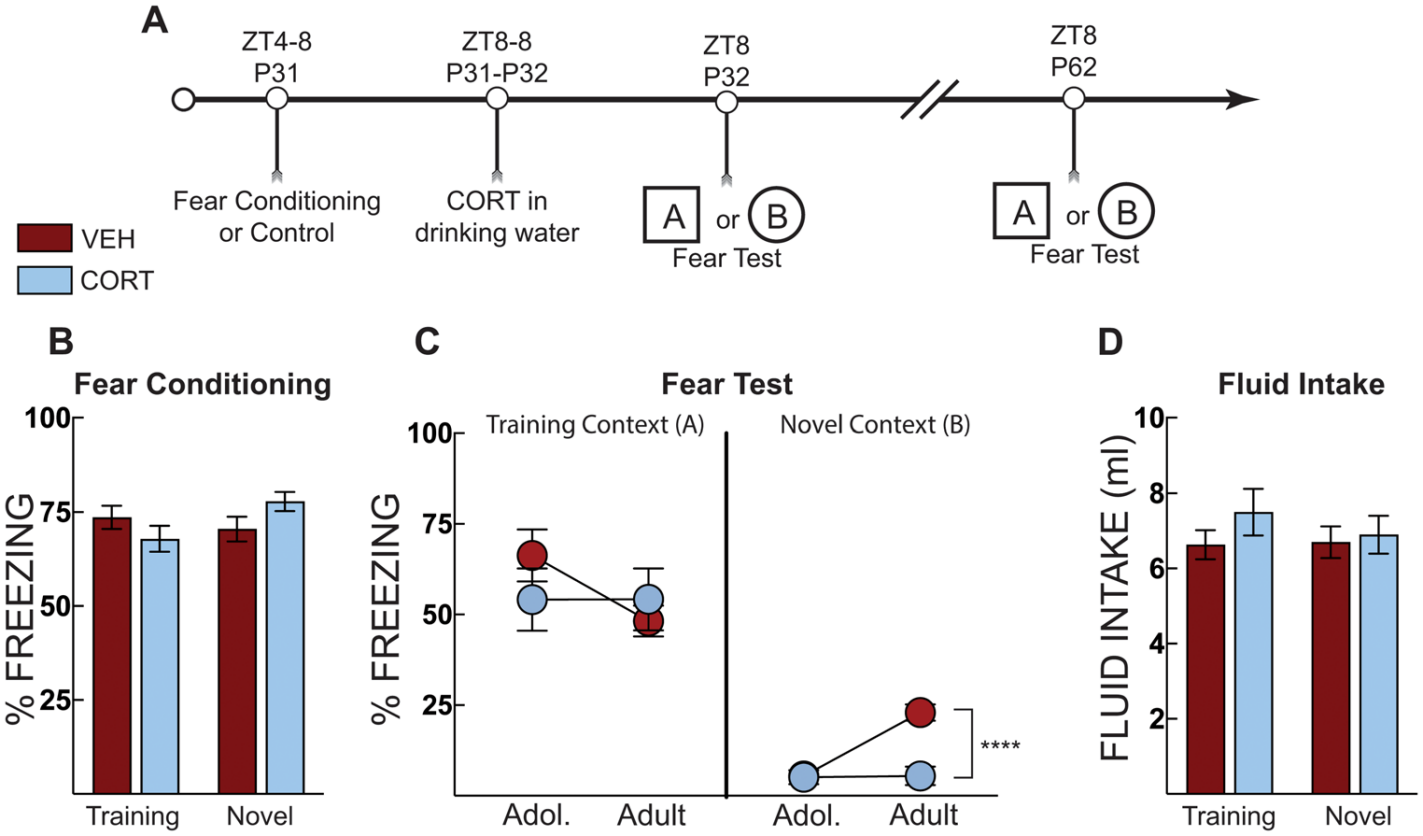
Post stress CORT prevents fear generalization into adulthood. (**A**) Timeline for context fear training and CORT treatment on fear expression in the training context [A] or a novel context [B]. Mice that were tested in the training context in adolescence (P32) were tested in the training context as adults (P62). The same procedure was applied to those mice tested in the novel context. (**B**) Context fear acquisition of vehicle (VEH, Red Bars) and corticosterone (CORT, Blue Bars)-treated mice in their “to-be” testing contexts. There was no difference in fear acquisition (average of post-shock freezing across the 5 shocks) (**C**) Context fear expression as percent time spent freezing over three minutes in the training context or in a novel context in adolescence (P32) and adulthood (P62). No differences were observed between VEH- and CORT-treated mice in the training context. However, CORT-treated mice displayed lower freezing compared to VEH-treated mice when tested in the novel context. VEH-treated mice demonstrate a time-dependent increase in freezing when tested in the novel context, whereas CORT treatment blocks this increase. (**D**) Total fluid intake over the 24 h period, there was no effect of grouping on fluid consumption. VEH Training Context (*n* = 10), CORT Training Context (*n* = 9), VEH Novel Context (*n* = 10), CORT Novel Context (*n* = 10). **** = *P* < 0.0001; Two-way ANOVA with Sidak’s post hoc test

To further explore the fear maintenance effect across development we observed in the previous experiment (Fig. 2C), In Experiment 2b, we utilized a procedure in which mice were tested for fear expression in the original training context or a novel distinct context (Fig. 3A). Context fear training increased freezing for all groups (Fig. 3B). Our main goal in this experiment was to determine if CORT treatment influenced freezing in the novel context. Thus, we ran a two-way repeated measures ANOVA on freezing in the novel context (treatment x age factors). Although not directly compared, overall, mice tested in the training context exhibited more freezing compared to those tested in the novel context (Fig. 3C). CORT administration had different effects on freezing in the novel context (main effect of treatment: F[1,18] = 9.8, *p* = 0.006; main effect of age: F[1,18] = 50.44, *p* < 0.0001; treatment x age interaction: F[1,18] = 46.65, *p* < 0.0001). During the adolescent test, both vehicle-treated and CORT-treated mice displayed very low levels of freezing in the novel context. However, during the adult test, vehicle-treated mice displayed increased freezing compared to CORT-treated mice (*p* < 0.0001, Šidák’s MC test; Fig. 3C). Again, there were no effects of treatment or shock exposure on fluid intake (Fig. 3D). The same pattern of results was observed across age and treatment when mice were tested in the training context as in the previous experiment (Fig. 2C). Vehicle-treated mice reduced freezing with age, but these effects were not statistically significant (two-way repeated ANOVA). Of note, while vehicle-treated mice increased freezing levels in the novel context, these levels are substantially lower than typical levels reported for fear in novel contexts at remote time points in adult mice (Cullen et al. 2015; Jasnow et al. 2012, 2017; Ortiz et al. 2019; Wiltgen and Silva 2007; Zhou and Riccio 1996). These results suggest that corticosterone may maintain long-term memory specificity during adolescent development and reduce generalization.

### Experiment 3: post-stress CORT blocks morphine place preference in adult mice, and incubation of morphine place preference in adolescent mice

**Fig. 4:**
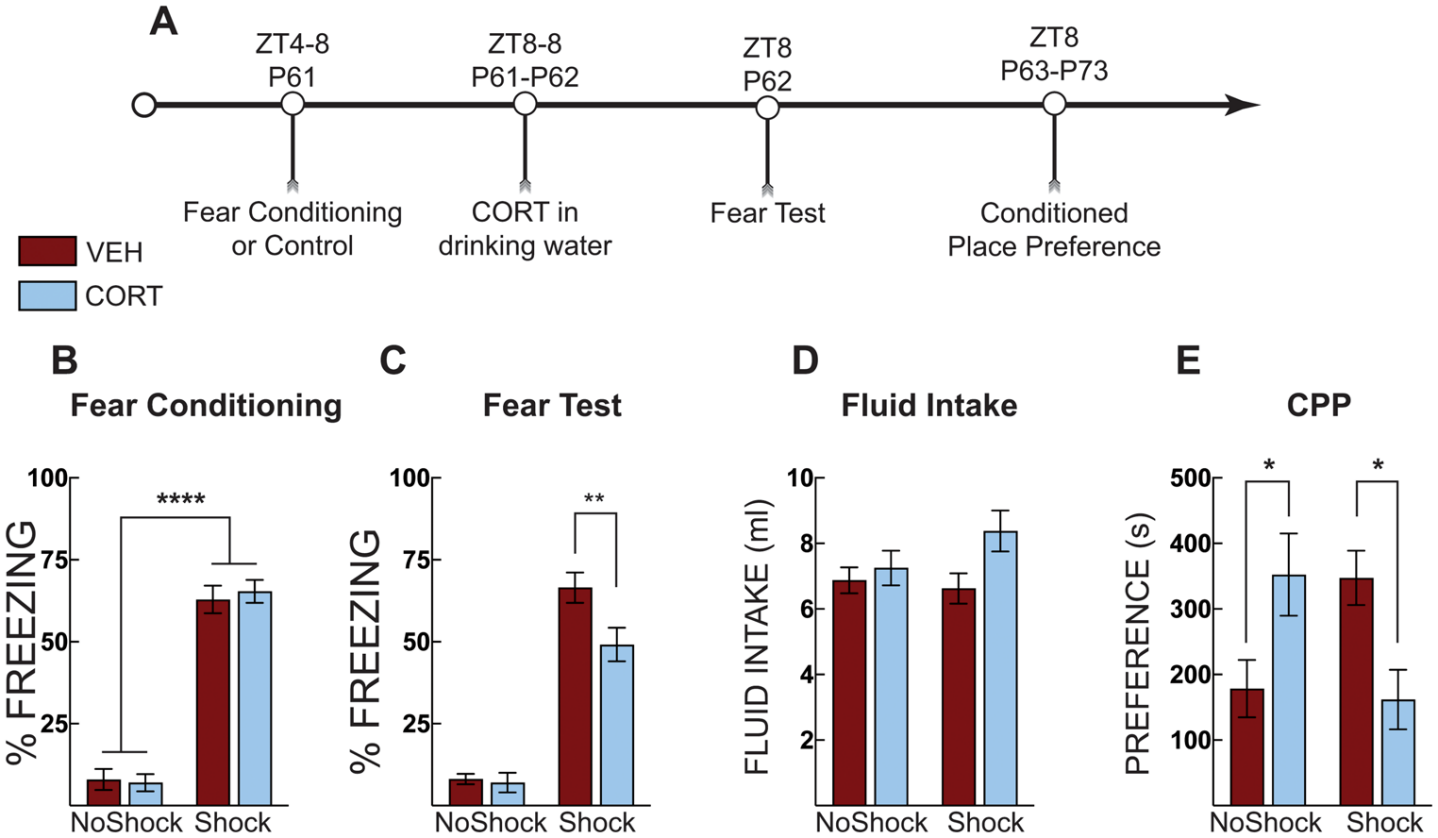
Post-stress CORT blocks morphine place preference in adults. (**A**) Timeline for adult context fear conditioning and corticosterone (CORT) treatment on fear expression and morphine place preference. (**B**) Freezing during context fear conditioning. There was no difference in fear acquisition (average of post-shock freezing across the 5 shocks) between vehicle-treated (VEH, Red Bars) and corticosterone (CORT, Blue Bars)-treated groups. Shocked mice displayed increased freezing compared to no shock control mice. (**C**) Context fear expression as percent time spent freezing over three minutes. Context fear conditioned mice that were administered CORT displayed less freezing compared to VEH-treated mice. Shocked mice displayed greater freezing compared to no shock control mice regardless of treatment. (**D**) Total fluid intake over the 24 h period, there was a main effect of treatment on fluid intake; CORT-treated mice drank more water than VEH-treated mice, but no individual post hoc comparison was significant. (**E**) CORT administration or shock increased morphine conditioned place preference. CORT-administration in shocked mice prevented potentiated morphine place preference. No shock VEH (*n* = 7), No shock CORT ( *n* = 7), Shock VEH (*n* = 7), Shock CORT (*n* = 6). * = *p* < 0.05; ** *p* < 0.01; **** *p* < 0.0001; Two-way ANOVA with Sidak’s post hoc test

In Experiment 3a, to test if CORT administration blocks stress-induced effects in adult mice, thirty-two adult mice were exposed to context fear conditioning as a foot shock stress (P62) and then administered CORT in the drinking water (Fig. 4A). Three mice were removed from the analysis as behavioral outliers. Fear-conditioned mice acquired fear, as evidenced by increased freezing compared to no shock control mice (main effect of shock: F [1, 27] = 263, *p* < 0.0001; Fig. 4B). When tested for fear, CORT administration reduced freezing for the fear-conditioned mice (main effect of shock: F [1, 26] = 170; *p* < 0.001, main effect of treatment F [1, 26] = 5.73, *p* = 0.024, treatment X shock interaction: F [1, 26] = 4.46, *p* = 0.044; Fig. 4C). CORT-treated mice had a slight increase in total fluid intake (CORT or VEH fluid intake) (main effect of treatment: F [1, 28] = 4.351, *p* = 0.046; Fig. 4D).

The day after fear testing, the mice underwent the morphine place preference procedure (Fig. 4A). Context fear conditioning potentiated morphine place preference for adult mice; however, CORT reduced this preference for fear-conditioned mice only (treatment X shock interaction: F [1, 23] = 13.1, *p* = 0.001; Fig. 4E). CORT treatment again potentiated morphine place preference in the absence of stress (*p* = 0.037). On the other hand, fear-conditioned mice given CORT had morphine place preference scores significantly lower than those given vehicle (*p* = 0.032). These data suggest that CORT treatment rapidly prevents stress-induced morphine place preference in adult mice. We also demonstrate that CORT treatment after context fear significantly reduces fear memory, unlike what we observe in adolescence.

**Fig. 5:**
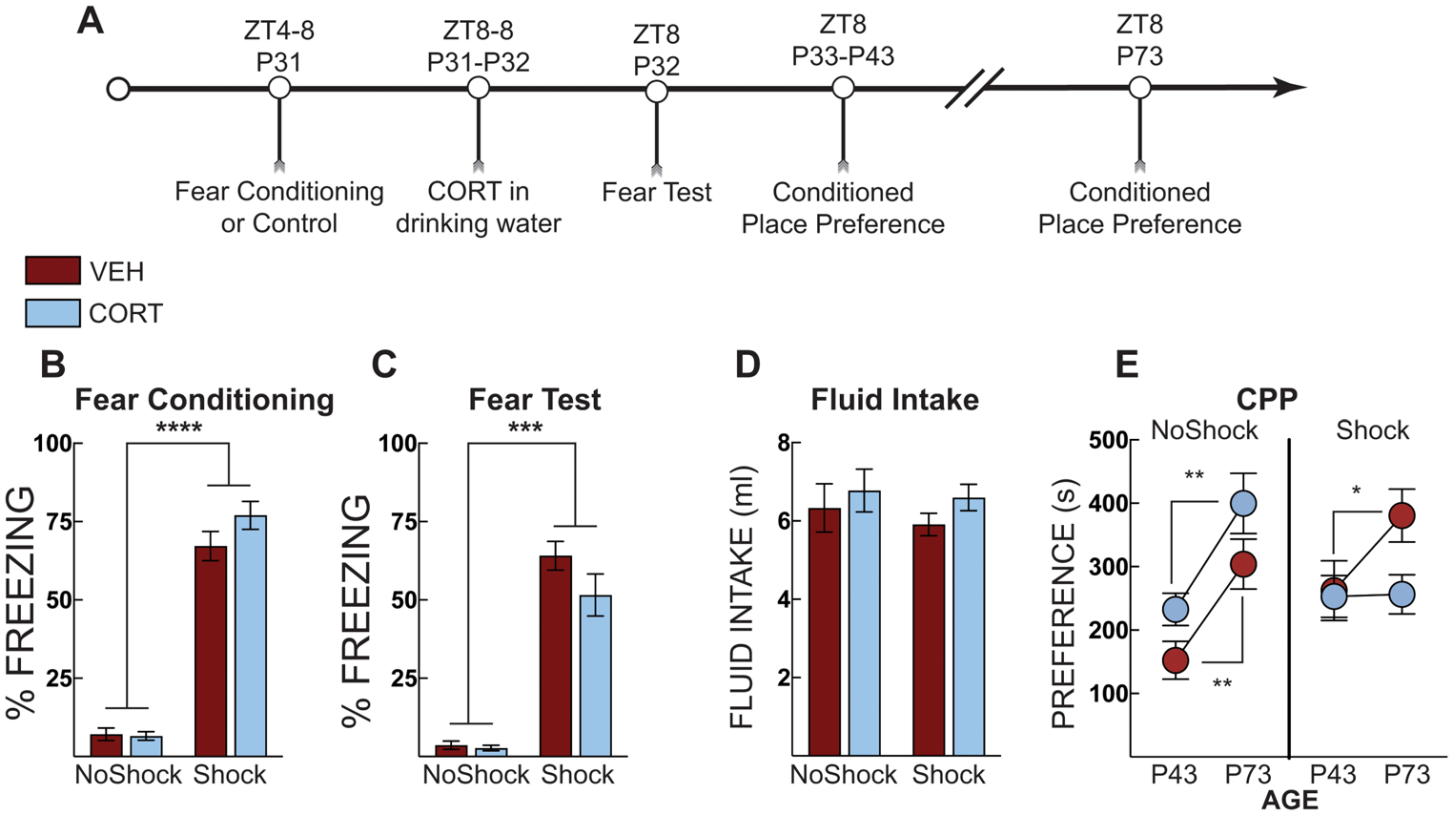
Post stress CORT in adolescence blocks incubation of morphine place preference. (**A**) Timeline for adolescent context fear conditioning and incubation of morphine place preference. (**B**) Freezing during context fear conditioning. There was no difference in fear acquisition (average of post-shock freezing across the 5 shocks) between vehicle-treated (VEH, Red Bars) and corticosterone (CORT, Blue Bars)-treated groups. Shocked mice displayed increased freezing compared to no shock control mice. (**C**) Context fear expression in adolescence as percent time spent freezing over three minutes. Shocked mice diplayed more freezing than no shock controls but there were no differences across treatment groups. (**D**) Total fluid intake over the 24 h period. (**E**) Morphine conditioned place preference in adolescence showed that CORT does not reduce preference for the morphine paired side in adolescence. However, CORT treatment blocked incubation of preference – all groups increased their preference scores when tested in adulthood except for the CORT-treated mice that received shock. No shock VEH (*n* = 12), No shock CORT (*n* = 9), Shock VEH (*n* = 10), Shock CORT (*n* = 11). **** *P* < 0.0001; *** *P* < 0.001 ** = *P* < 0.01; * = *p* < 0.05; Two-way ANOVA with Sidak’s post hoc test and Three- way ANOVA with Sidak’s in (E)

In Experiment 3b, our aim was to identify if CORT administration reduced stress-potentiated morphine place preference rapidly in adolescence. Forty-two adolescent mice were exposed to context fear conditioning and then given CORT until context fear testing the next day (Fig. 5A). Fear-conditioned mice acquired fear, as evidenced by increased freezing compared to no shock control mice (main effect of shock: F [1, 38] = 388.2, *p* < 0.0001] (Fig. 5B). Fear conditioning produced higher freezing compared to controls (main effect of shock F [1,38] = 177, *p* < 0.001]; Fig. 5C). There were no differences between groups for total CORT or vehicle fluid intake (Fig. 5D). Adolescent mice then underwent morphine place preference conditioning and a place preference test on P43. CORT treatment did not rapidly prevent morphine-conditioned place preference in adolescent mice. Mice were left undisturbed until adulthood, when they underwent a second morphine preference test 30 days later (P73). We ran a repeated measure three-way ANOVA using shock, treatment, and age as variables with a Šidáks multiple comparison test across treatment and age. All groups except fear-conditioned (shocked) mice that received CORT increased their preference scores compared to their adolescent score (main effect of age: F [1, 38] = 35.10; *p* < 0.0001; age x shock interaction: F [1,38] = 7.01, *p* = 0.012; and a shock x treatment interaction: F [1,38] = 5.63, *p* = 0.023; Fig. 5E). These data indicate incubation of morphine place preference over 30 days and across a developmental transition, which is prevented by a single post-stress CORT treatment in adolescence.

### Experiment 4: Post-stress CORT blunts serum corticosterone but is unrelated to freezing

**Fig. 6:**
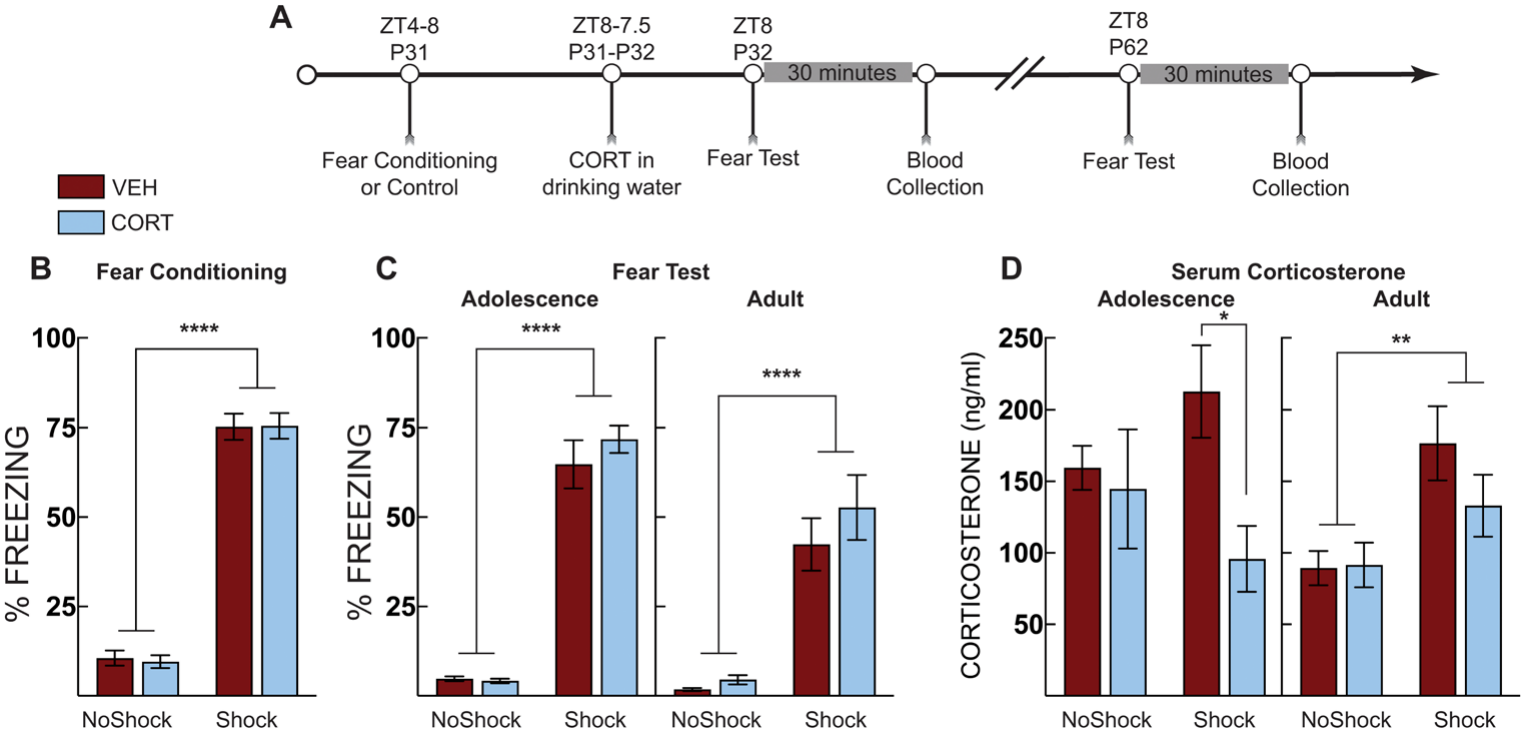
Post stress CORT blunts serum corticosterone. (**A**) Timeline for adolescent fear conditioning and serum corticosterone analysis. (**B**) Freezing during context fear conditioning. There was no difference in fear acquisition (average of post-shock freezing across the 5 shocks) between vehicle-treated (VEH, Red Bars) and corticosterone (CORT, Blue Bars)-treated groups. Shocked mice displayed increased freezing compared to no shock control mice. (**C**) Context fear expression in early adolescence (P32; Left) and adulthood (P62; Right). At both tests, shocked mice displayed more freezing than no shock controls but there were no differences across treatement groups. (**D**) Serum corticosterone during adolescence (P32; Left) and adulthood (P62; Right). In adolescence, serum corticosterone was suppressed in shocked mice that were administered CORT compared to VEH-treated mice. In adulthood, serum corticosterone was elevated in shocked mice compared to no shock controls regardless of treatment. No shock VEH (*n* = 10 P32; *n* = 6 P62), No shock CORT ( *n* = 9 P32; *n* = 6 P62), Shock VEH (*n* = 6 P32; *n* = 6 P62), Shock CORT (*n* = 6 P32; *n* = 6 P62). Two-way ANOVA with Sidak’s post hoc test; * *p* < 0.05; ** *p* < 0.01; **** *p* < 0.0001

To identify processes mediating the interaction between stress exposure and corticosterone on fear expression testing, adolescent mice were exposed to contextual fear conditioning followed by CORT in their drinking water for 24 h. Mice were tested for freezing as adolescents (P32) or adults (P62). Thirty minutes after each test, mice were euthanized, and blood was taken for corticosterone analysis (Fig. 6A). Fear-conditioned mice acquired fear, as evidenced by increased freezing compared to no shock control mice (main effect of shock: F [1, 48] = 541.8, *p* < 0.0001; Fig. 6B). During the fear test in early adolescence (P32), shocked mice displayed elevated freezing compared to no-shock controls (main effect of shock: F [1, 28] = 454.7, *p* < 0.0001; Fig. 6C), but there were no differences between vehicle-treated and CORT-treated mice in freezing. At the adult (P62) test, fear-conditioned mice, again, showed significantly elevated freezing compared to no-shock control mice (main effect of shock: F [1, 20] = 57.25, *P* < 0.0001; Fig. 6D), but there was no difference between treatment groups. In adolescence, CORT-treated mice, however, had reduced serum corticosterone compared to vehicle-treated mice (main effect of treatment: F [1, 26] = 4.887, *P* = 0.036; Fig. 6D). This effect was limited to fear-conditioned (shocked) mice (Šidák MC test, *p* = 0.034), and not observed in no-shock controls. As adults, fear-conditioned (shocked) mice had increased serum corticosterone compared to no-shock controls (main effect of shock: F [1, 20] = 10.77, *p* = 0.0037; Fig. 6D). In adulthood, fear-conditioned mice did not display significant differences in serum corticosterone compared to shocked vehicle controls. There were also no differences between groups in total CORT or vehicle fluid intake (Veh-No shock: 9.9 ±1.2; CORT-No Shock: 11.5 ±1.5; Veh-Shock: 9.6±1.5; CORT-Shock: 9.7±1.2; Interaction effect F [1,49] = 0.3288; *p* = 0.57).

## Discussion

The findings from these experiments provide new evidence that a single overnight administration of CORT can prevent the long-term effects of adolescent stress. The current literature shows that CORT administration in adults prevents the delayed effects of restraint or immobilization stress on anxiety-like behavior, social behavior, and amygdala spine density 10 days later (Chakraborty et al. 2020; Rao et al. 2012). Here, we extend those findings and demonstrate that post-stress corticosterone administration in early adolescence prevents the long-term effects on adult behaviors and includes social behavior, fear expression, and drug-associated place preference. These findings also demonstrate adolescent corticosterone treatment has delayed effects on social interaction and morphine place preference, which do not emerge until adulthood. Moreover, we show the protective effects of corticosterone treatment can extend to 40 days after adolescent stress exposure.

We provide additional support for and extend prior work on the combination of stress and CORT administration, negating the effects of either treatment alone—an effect that has been considered a paradox ever since its first reporting (Chakraborty et al. 2020; Magarinos et al. 1998; Rao et al. 2012). A more recent study found the effects of CORT treatment were replicated even if it was administered 24 h after restraint stress (Chakraborty et al. 2020). Exogenous CORT 24 h after restraint stress increased social interaction, but when administered alone, it decreased social interaction, similar to our results (Fig. 1D).

In our first experiment, CORT administered to defeated mice produced no effects in early adolescence but increased interaction comparable to non-defeated control mice in adulthood. This suggests that CORT administration promotes resilience to adolescent social stress and normalizes maladaptive social behavior in adulthood. All mice decreased social interaction times as they aged, with mice spending more time interacting with a novel mouse during their adolescent test than they did in adulthood. Given that adolescence is marked by increased social exploration and novelty seeking, the result that adolescent mice interact at higher levels than adult mice is in line with the adolescent literature (Spear 2000). When measuring morphine place preference, defeated mice had potentiated place preference compared to non-defeated mice. This corroborates other findings using unpredictable stress in adolescence (Wang et al. 2019). CORT treatment normalized the stress-potentiated morphine place preference to levels comparable to non-defeat controls. However, CORT administration to non-defeat adolescent controls also potentiated morphine place preference. This interaction suggests that social defeat or a single overnight administration of CORT in early adolescence has long-term impacts on drug-conditioned place preference 40 days after the defeat or CORT administration.

We next used context fear conditioning as a stressor to determine if the effects of corticosterone on social behavior and morphine place preference replicated with a different stress procedure. As the mice aged, there was a significant reduction in freezing for context fear-conditioned mice that received vehicle (Fig. 2C). Like the social defeat experiment, CORT treatment prevented the stress-potentiated morphine place preference, and this was not due to anhedonia (Fig. 2E, F). Whether the above-mentioned reduction in freezing across time was due to forgetting (Hardt et al. 2013; Tzakis et al. 2020) or rapid extinction (Myers and Davis 2006) caused by the 24-hour adolescent test is unclear. The reduction in freezing was not observed in context fear-conditioned mice that received CORT. Exogenous CORT may promote context memory retention and block reduced fear across time due to forgetting or rapid extinction that may have occurred in adolescence during the fear test. To address this, we exposed a subsequent cohort of mice to context fear tests in the training context or a novel context (Fig. 3). When mice were tested in the novel context, vehicle-treated mice showed increased freezing compared to CORT-treated mice when tested as adults (Fig. 3C). We interpret these results as CORT promoting maintenance of context memory specificity across development and suppressing generalization. Thus, the reduction of freezing we observed across time is not likely due to rapid extinction but may instead be related to forgetting. Again, we note that the amount of freezing in the novel context during the adult test, although elevated in vehicle-treated mice compared to CORT-treated mice, is much lower than that reported in previous literature examining generalized fear in adults (Cullen et al. 2014, 2015; Lynch et al. 2017; Ortiz et al. 2019). It is important to note, however, that we do not have a corresponding 30-day test of generalization in adults to determine if these effects are due to time or an interaction between time and a developmental transition.

To test the immediate effects of corticosterone in adolescence, mice were trained for context fear conditioning and then administered CORT in the drinking water until their contextual fear test. The day after the context fear test, mice began the morphine place preference paradigm and were tested 30 days after the conditioned place preference test again (Fig. 5E). All mice, except for those exposed to shock and CORT in adolescence, increased morphine place preference from adolescence to adulthood. This suggests significant incubation of morphine place preference, a non-contingent measure of increased craving (Bardo and Bevins 2000; Tzschentke 2007), and that CORT blocks this incubation. CORT administration did not have an immediate effect on reducing potentiated preference scores in adolescents. Notably, when adult mice underwent morphine-conditioned place preference, the effects of corticosterone treatment were observed immediately (Fig. 4E), suggesting a developmental dissociation in the effectiveness of CORT between adolescents and adults. Furthermore, when CORT was administered to adult mice after shock exposure, it reduced freezing during the context test the next day (Fig. 4C). However, the same treatment in adolescent mice had no immediate effect on freezing (Figs. 2, 3 and 6); it appeared to maintain fear to the training context in which the shock occurred but reduced freezing to a novel context in adulthood (Figs. 2 and 3). We also did not observe significant potentiation of morphine CPP in response to shock or CORT-treatment when mice were trained and tested in adolescence. This is different than what we observed when mice underwent stress in adolescence and tested in adulthood (Fig. 2D versus Fig. 5E). Previous literature has demonstrated that post-training CORT enhances fear memory (Hui et al. 2004; Roozendaal et al. 2004), at odds with the effects observed in Fig. 5C in adults. This could be due to differences in administration; previous experiments used subcutaneous injections, and there are known differences in effects between subcutaneous and water-administered CORT (Rao et al. 2012). CORT administration significantly suppressed serum corticosterone in shock-exposed adolescent mice, and although serum corticosterone was also lower in adults, the effect in adults was not significant (Fig. 6). Serum corticosterone in the No Shock group is slightly higher than we have measured at baseline in adolescent mice (~ 100 ng/ml) (Latsko et al. 2016), suggesting that being placed in the conditioning chamber mildly increases corticosterone release. Importantly, serum corticosterone was not beyond the physiological range in CORT-treated mice. Thus, the effects of CORT administration were stronger in adolescent mice, at least on serum corticosterone, yet did not immediately reduce morphine-conditioned place preference or alter fear responses as it did in adults. Unfortunately, we do not have the adult comparison group to compare the corticosterone response directly, but based on previous work in adult rodents, we predict similar serum corticosterone-suppressing effects of CORT administration (Chakraborty et al. 2020). The findings of these experiments suggest that CORT reduces morphine place preference, in part, by the transition from adolescence to adulthood and is not simply the result of time between treatment and testing. If the effects of CORT interact with time and not the transition from adolescence to adulthood, then CORT treatment in adult mice would not have alleviated stress-potentiated preference for a morphine-associated context immediately and would have emerged only after a delay. Although still debated, low circulating cortisol - the primary glucocorticoid in humans - soon after trauma has been proposed as a risk factor for the development of PTSD (Hruska et al. 2014; Yehuda and LeDoux 2007; Yehuda et al. 2014). Children tend to show the same pattern of low cortisol levels but with a delay following trauma exposure (Goenjian et al. 1996; King et al. 2001). Although trauma exposure during youth substantially elevates adult risk for mood and anxiety disorders as well as substance abuse (Chapman et al. 2004; Dube et al. 2001; Felitti et al. 1998; Gladstone et al. 2004; Jasnow and Ressler 2006; McCauley et al. 1997), it is not entirely clear how this happens or why some individuals are more vulnerable than others. Adolescent social defeat results in adult susceptible and resilient social behavior phenotypes (Latsko et al. 2016). Moreover, resilient mice exhibited elevated endogenous corticosterone during an adolescent social interaction test (Latsko et al. 2016). Regardless of the adult behavioral phenotype, endogenous corticosterone was positively correlated with adult social interaction levels in defeated mice but not in the control group. These data suggest a relationship among stress, adolescent HPA axis responsiveness, and adult socioemotional behavior phenotypes. In the current study, we did not divide individual responses into behavioral phenotypes; instead, we chose to examine behavior as a continuous variable, especially because our earlier analysis showed that endogenous corticosterone in defeated mice was associated with social approach behavior regardless of the artificially designated behavioral phenotype (Latsko et al. 2016), though there is individual variability in the response.

Serum corticosterone after CORT treatment in adolescence was not elevated after fear conditioning, nor did it correlate with fear expression. Specifically, CORT-treated mice had similar serum corticosterone levels regardless of shock exposure. The blunted corticosterone was not associated with lower levels of fear expression either. CORT-treated mice displayed high levels of freezing in adolescence compared to vehicle-treated mice. CORT-treatment likely suppressed endogenous corticosterone release due to its actions at the pituitary or hypothalamus, though we don’t have measurements to make definitive conclusions. As adults, CORT-treated mice did show lower freezing and lower serum corticosterone, but these effects were not statistically different from vehicle-treated mice that also underwent stress as adolescents. These results suggest that serum corticosterone levels are not indicative of fear expression, at least in adolescence, and that future studies that use corticosterone measures to indicate heightened stress levels should be interpreted carefully. Age and individual differences may drive the release of corticosterone over behavioral testing, or it may reflect differences in metabolic rate (Jimeno and Verhulst 2023). The actions of CORT treatment on adult CPP may instead be through direct actions on glucocorticoid receptors in the hippocampus, prefrontal cortex, or nucleus accumbens, three regions involved in drug-conditioned place preference. Our previous work on social avoidance after adolescent mice undergo social defeat suggests that the actions of exogenous CORT are through glucocorticoid receptors (Latsko 2018) but this was not localized to a specific brain region. However, we have not directly tested the role of glucocorticoid receptors in mediating the effects of CORT on CPP. Glucocorticoid receptors within the basolateral amygdala mediate the effects of chronic exogenous CORT on elevated fear expression (Conrad et al. 2004), further supporting the idea that glucocorticoid receptors mediate the behavioral effects observed here. However, mineralocorticoid receptors in the infralimbic cortex mediate stress-induced fear extinction impairments (Albernaz-Mariano and Demarchi Munhoz 2022). Therefore, we cannot absolutely rule out the role of mineralocorticoid receptors in mediating the effects of CORT on CPP here.

The mechanism for the paradox of exogenous CORT providing benefits after stress exposure remains unidentified. Previous studies have focused on the effect of stress plus CORT administration on pyramidal neuron spine density in the amygdala, but these studies have only examined the physiology ten days after stress and CORT administration (Chakraborty et al. 2020; Rao et al. 2012). How the increased spine density in stressed rodents directly contributes to elevated anxiety-like behavior is not established, but there is a correlation between spine density and anxiety-like behavior in those studies. The effect, however, is unlikely to be due to the increased corticosterone levels due to the stress itself because when exogenous CORT is given in the absence of stress, it mimics the effect of stress alone, and this agrees with previous literature (Chakraborty et al. 2020). One possibility is that two surges of corticosterone alter molecular signaling or methylation and expression of the GR-chaperone FKBP5 (Galatzer-Levy et al. 2013; Sawamura et al. 2016), inducing differential downstream genomic effects of GR-initiated transcription. Another possibility is that because CORT was administered four hours before the dark phase of the light: dark cycle, disruption of the natural corticosterone diurnal rhythm could initiate long-term molecular changes that reverse the effects of stress alone because most intake occurs after lights out. However, Chakraborty et al. (2020), administered CORT in the drinking water during most of the day and found similar prophylactic effects. Thus, it is unlikely to be a single overnight disruption of the normal corticosterone diurnal rhythm that causes such long-term effects, though this has not been explicitly examined. Alternatively, it is well-established that juvenile exposure to acute stress procedures increases adrenocorticotropic hormone (ACTH) and corticosterone secretion that persists for twice as long compared with adult rats (Goldman 1973; Romeo 2003; Romeo et al. 2008; Romeo et al. 2006; Romeo et al. 2016). Prepubertal rats also have greater corticotropin-releasing hormone (CRH) expression in the PVN after stress compared to adults. Exogenous CORT exposure could disrupt this endogenous ACTH and corticosterone secretion in response to stress and provide a mechanism for the protective effects of exogenous CORT. As noted above, however, the endogenous corticosterone appeared unrelated to behavioral freezing during fear memory tests, suggesting a dissociation between endogenous corticosterone and behavior. Despite displaying elevated freezing, the adolescent mice that were administered CORT exhibited endogenous corticosterone levels similar to no-shock control adult mice. Thus, while exogenous CORT did not change behavior, it significantly altered the HPA-axis response. In conclusion, our results are translationally relevant for understanding how early life stress impacts stress-responsive and appetitive behavior into adulthood, and we identify important interactions between stress exposure, development, and glucocorticoid regulation. CORT administration has been demonstrated to protect against the anxiety-like behavior induced by restraint stress for up to 10 days in adult rodents (Chakraborty et al. 2020). Here, we show that CORT administration prevents stress-potentiated reward-like behavior for up to 40 days when mice are adults but does not have immediate benefits in adolescence. Further, our study supports the idea that hydrocortisone can be a useful post-stress prophylactic agent but must be used under specific stress conditions. Glucocorticoids have been used with some success in adults to reduce trauma-induced PTSD symptoms (Aerni et al. 2004; Delahanty et al. 2013; Hruska et al. 2014; Schelling et al. 2001, 2004; Suris et al. 2010). Importantly, exogenous glucocorticoids reduce the response to trauma reminders in patients with PTSD, suggesting it has lasting effects on PTSD symptoms (Suris et al. 2010). We show that corticosterone can be a clinically effective therapeutic agent for preventing the long-term impacts of adolescent stress and find novel protection against stress-potentiated reward behavior that broadens the impact of using hydrocortisone therapeutically.
